# Molecular Characterization, mRNA Expression and Alternative Splicing of Ryanodine Receptor Gene in the Brown Citrus Aphid, *Toxoptera citricida* (Kirkaldy)

**DOI:** 10.3390/ijms160715220

**Published:** 2015-07-06

**Authors:** Ke-Yi Wang, Xuan-Zhao Jiang, Guo-Rui Yuan, Feng Shang, Jin-Jun Wang

**Affiliations:** Key Laboratory of Entomology and Pest Control Engineering, College of Plant Protection, Southwest University, Chongqing 400715, China; E-Mails: may344026835@163.com (K.-Y.W.); jxzzby@163.com (X.-Z.J.); ygr@swu.edu.cn (G.-R.Y.); fengshang1994@126.com (F.S.)

**Keywords:** *Toxoptera citricida*, ryanodine receptor, mRNA expression, alternative splicing

## Abstract

Ryanodine receptors (RyRs) play a critical role in regulating the release of intracellular calcium, which enables them to be effectively targeted by the two novel classes of insecticides, phthalic acid diamides and anthranilic diamides. However, less information is available about this target site in insects, although the sequence and structure information of target molecules are essential for designing new control agents of high selectivity and efficiency, as well as low non-target toxicity. Here, we provided sufficient information about the coding sequence and molecular structures of RyR in *T. citricida* (TciRyR), an economically important pest. The full-length TciRyR cDNA was characterized with an open reading frame of 15,306 nucleotides, encoding 5101 amino acid residues. TciRyR was predicted to embrace all the hallmarks of ryanodine receptor, typically as the conserved C-terminal domain with consensus calcium-biding EF-hands (calcium-binding motif) and six transmembrane domains, as well as a large N-terminal domain. qPCR analysis revealed that the highest mRNA expression levels of TciRyR were observed in the adults, especially in the heads. Alternative splicing in TciRyR was evidenced by an alternatively spliced exon, resulting from intron retention, which was different from the case of RyR in *Myzus persicae* characterized with no alternative splicing events. Diagnostic PCR analysis indicated that the splicing of this exon was not only regulated in a body-specific manner but also in a stage-dependent manner. Taken together, these results provide useful information for new insecticide design and further insights into the molecular basis of insecticide action.

## 1. Introduction

Intracellular calcium (Ca^2+^) homeostasis is crucial for normal life activities because Ca^2+^ is a key second messenger that regulates a number of cellular processes, such as muscle contraction, hormone secretion, synaptic transmission, and gene transcription [1]. Ryanodine receptors (RyRs), also known as Ca^2+^ release channels, play an critical role in intracellular Ca^2+^ signaling by inducing the release of Ca^2+^ from the sarco/endoplasmic reticulum (SR/ER) lumen to the cytosol in muscle and non-muscle cells [2,3]. RyRs are homomeric tetramers with a total molecular mass higher than 2 MDa [4], composed of a hydrophilic domain at the N-terminus and a membrane-spanning domain at the C-terminus [5]. The N-terminal cytoplasmic domain, also known as the “foot structure”, is large and spans the junction between the transverse tubules and SR membranes. The C-terminal hydrophobic domain contains 4–12 transmembrane segments and forms the putative pore of the Ca^2+^ release channel [4,6]. In mammals, three RyR isoforms have been reported: RyR1, which is predominant in skeletal muscles, RyR2 is largely present in cardiac muscles, and RyR3, which is primarily present in the brain and diaphragm [6,7]. However, in birds, amphibians, and fish only two RyR isoforms (RyRA and RyRB), which are closely related to the mammalian RyR1 and RyR3, have been identified [8,9]. Unlike the vertebrates, insects are known to have only one RyR coding gene (*Rya-r44F*) [10], which shares about 45% sequence identity with either of the three mammalian RyRs [11].

Because the remarkable difference between the insects and mammalian RyRs could minimize the non-target effects on mammals, insect RyRs have been used as targets for insecticides, particularly phthalic acid diamides and anthranilic diamides [12,13]. These insecticides were previously known to be highly effective to lepidopteran pests [14], while recent researches indicate that these insecticides are also effective in controlling whiteflies and oriental fruit flies [15,16]. Hence, it is useful to estimate the toxicity of diamide insecticides to other insects in addition to lepidopteran pests, which will put forward the development of RyR targeting insecticides. In addition, the coding sequences of RyRs may provide key information to understand the molecular base that confers the high selectivity of these diamide insecticides. However, despite the increased availability of insect genomes, only 15 full-length insect RyR cDNAs have been cloned thus far. These RyRs are from insects including *Drosophila melanogaster* (*DmRyR*), *Cnaphalocrocis medinalis* (*CmRyR*), *Nilaparvata lugens* (*NlRyR*), *Bemisia tabaci* (*BtRyR*), *Chilo suppressalis* (*CsRyR*), *Laodelphgax striatellu*s (*LsRyR*), *Bactrocera dorsalis* (*BdRyR*), *Plutella xylostella* (*PxRyR*), *Ostrinia furnacalis* (*OfRyR*), *Helicoverpa armigera* (*HaRyR*), *Pieris rapae* (*PrRyR*), *Myzus persicae* (*MpRyR*), *Leptinotarsa decemlineata* (*LdRyR*), *Sogatella furcifera* (*SfRyR*) and *Tribolium castaneum* (*TcRyR*) [10,17,18,19,20,21,22,23,24,25,26,27,28]. It is known that diamides activate insect RyRs, the resistance could result in gene mutations in RyRs [29]. For example, high levels of diamide cross-resistance *P. xylostella* strains collected from Philippines, Thailand, and China were found to be over 200-fold more resistant to chlorantraniliprole compared with susceptible strains; this resistance feature was associated with a mutation (G4946E) in the C-terminal membrane-spanning domain of *PxRyR* [30,31]. Three novel mutations including E1338D, Q4595L, and I4790M were reported and displayed a significantly lower affinity to the chlorantraniliprole [32]. Hence, it is essential to obtain further insights into the molecular properties of insect RyRs.

Due to the importance of RyRs as an insecticide target and limited information available about insect RyRs, we sequenced the coding region of RyR from the brown citrus aphid, *Toxoptera citricida* (Kirkaldy) (Hemiptera: Aphididae), an efficient vector of *Citrus tristeza virus* (CTV), which has now colonized virtually all of the world’s citrus production areas. Comparative analysis indicated that RyR in *T. citricida* (TciRyR) is functional and structural orthologs of insect RyRs. Moreover, we confirmed that TciRyR was product of alternative splicing suggesting that insect RyRs obtain functional diversity by generating alternative splicing variants.

## 2. Results

### 2.1. Cloning the Coding Sequence of Ryanodine Receptor (RyR) in Toxoptera citricida (TciRyR)

**Figure 1:**
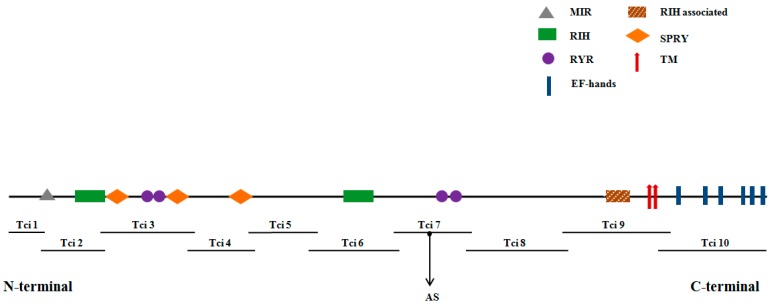
Analysis of conserved structural domains in ryanodine receptor (RyR) and the strategy used to clone RyR in *Toxoptera citricida* (TciRyR). All fragments labeled “Tci” were used to assemble TciRyR. Lines show the length and relative location of amplicons. The location of putative transmembrane segments and conserved structural domains predicted by the Conserved Domains Database (NCBI) is shown. These include: MIR (protein mannosyltransferase, IP_3_R and RyR domain), RIH (RyR and IP_3_R homology), SPRY (spore lysisA and RyR domains), RyR (RyR domain), and RIH-associated (RyR and IP_3_R homology associated) domains, and EF-hands (calcium-binding motif) and transmembrane segments. Arrows indicate the location of alternatively spliced exon.

A sequence of 15,639 bp was assembled from ten overlapping cDNA products (Figure 1), which covered the complete open reading frame (ORF) of 15,315 bp. The TciRyR (KP733849) ORF encoded a putative 5101-residues polypeptide, with a theoretical molecular weight of 580.08 kDa and PI (isoelectric point) of 5.49.

### 2.2. RyR Orthologs and Their Phylogeny

All RyR orthologs deposited in GenBank were investigated by using the key words “Ryanodine receptor” and “RyR”. The results showed that RyR orthologs were widely distributed in Chordata, Arthropoda, Nematoda, Platyhelminthes, Mollusca, Annelida, Echinodermata, Hemichordata, Placozoa, and Porifera, but not in Cnidaria, Protozoa, or Ctenophora. The Arthropoda orthologs with full-length cDNAs available were primarily found in Insecta, the majority of them are derived from Diptera, Lepidoptera, Hemiptera, Hymenoptera, and Coleoptera.

Multiple protein alignments showed that TciRyR shared high similarity with the known insect RyRs. TciRyR shared the highest similarity with the RyRs from *M. persicae* (98.2%) and *A. pisum* (98.1%) at the protein level, 92.5% and 93.1% at the nucleotide level, respectively. The similarity between TciRyR with other insect orthologs ranged from 70% to 90%, such as 74.7% with DmRyR from *D. melanogaster*, 76.3% with AgRyR from *A. gambiae*, 77.7% with sRyR from *B. mori* and 81.1% with NlRyR from *N. lugens*. The similarity between TciRyR with the three RyRs isoforms of *H. sapiens* ranged from 43.0% to 46.0%.

**Figure 2:**
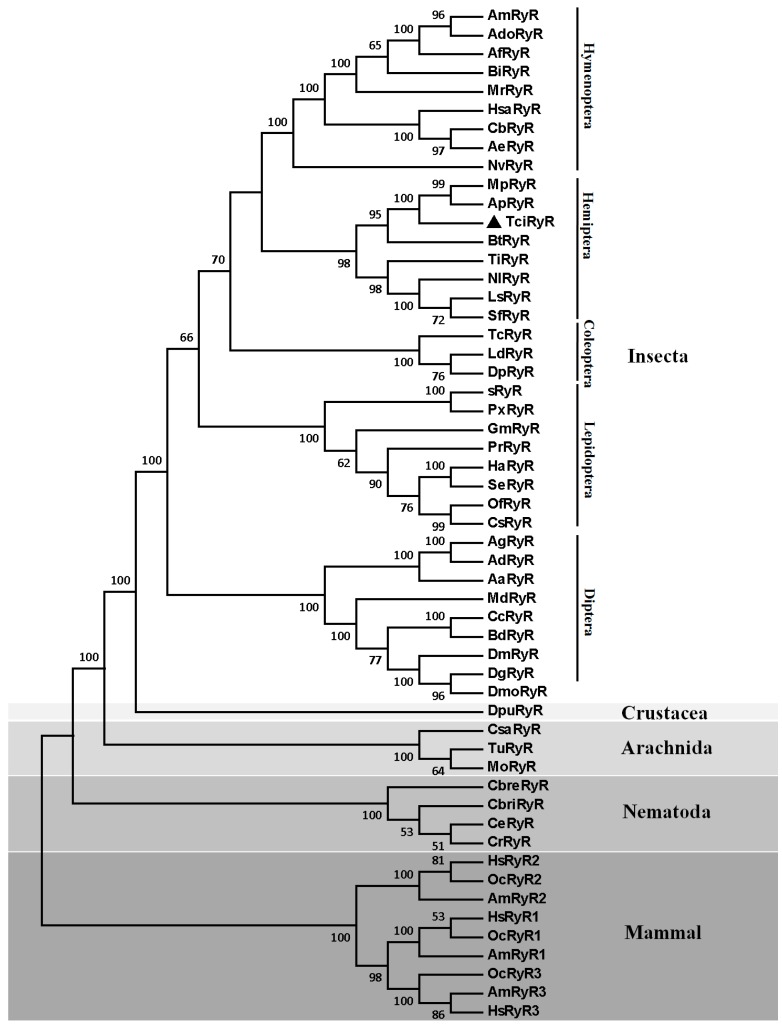
Phylogenetic tree of the ryanodine receptor (RyR) family. The TciRyR amino acid sequences were aligned to 53 representative RyR homologs from 47 species and used for phylogenetic analysis. Only bootstrap values exceeding 50% were shown at branch points. GenBank accession numbers of all sequences are listed in the Table S2.

The phylogenetic analysis using the protein sequences of TciRyR and 53 other RyR homologs, including 36 insect RyRs with complete ORFs, one crustacean RyR, three arachnid RyRs, four nematode RyRs, and nine vertebrate RyRs (Table S2) showed that TciRyR clustered with other hemipteran RyRs into a single branch (Figure 2). TciRyR was most close to the two aphid RyRs, MpRyR and ApRyR. Hemipteran RyRs were more close to the coleopteran and hymenoperan RyRs. Dipteran RyRs clustered into one single branch separated from all other insect RyRs. The homologs of all mammalian RyRs clustered into one branch, well separated from the RyRs of Nematodes, Arachnids, Crustaceans and Insects (Figure 2).

### 2.3. Conserved Structural Domains in TciRyR

The N-terminal region of TciRyR contains the mannosyltransferase, IP_3_R (Inositol 1,4,5-trisphosphate receptor), and RyR domains (MIR, pfam02815) between residues 212–393, two RyR and IP_3_R homology domains (RIH, pfam01365) at 440–648 and 2219–2450, respectively, three spore lysisA and RyR domains (SPRY, pfam00622) at positions 647–798, 1079–1211, and 1528–1679, respectively, four RyR repeat domains (pfam02026) at positions 852–946, 965–1059, 2829–2921 and 2946–3032, respectively, as well as an RIH-associated domain (pfam08454) at position 3985–4110 (Figure 1). Prediction of phosphorylation sites revealed the presence of three potential PKA (Protein kinase A) and ten potential PKC (Protein kinase C) sites in TciRyR. In addition, ten potential *N*-glycosylation sites were predicted in TciRyR.

**Figure 3:**
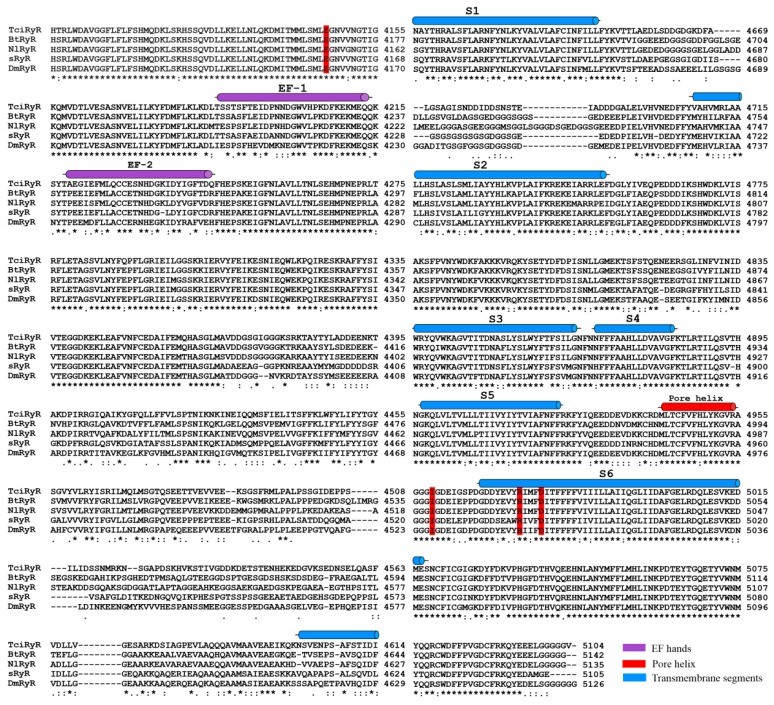
Multiple sequence alignment of the C-terminal region of insect RyR homologs. Numbers on the right indicate the amino acid position in each protein. An asterisk (*) denotes identical residues; double dots (:) represent a conserved residue substitution; a single dot (.) shows partial conservation of the residue. Secondary structural elements are indicated above the sequence alignment including two putative EF-hand motifs (EF1 and EF2), six transmembrane segments (S1 to S6) and a Pore-helix [33]. Important residues we discussed in the text (E^4145^, I^4958^, R^4974^, D^4978^) are shadowed in red. Abbreviations and GenBank entries for the RyR homologs are described in Table S2.

TciRyR contained all six hydrophobic transmembrane segments typical for RyRs (S1: 4445–4464, S2: 4634–4656, S3: 4710–4732, S4: 4855–4877, S5: 4900–4922, S6: 4980–4999, respectively) (Figure 3). The binding motif, GXRXGGGXGD, an essential part of the ryanodine binding and the channel conduction pathway, was located at 4951–4961 amino acid residues. The pore helix, which is analogous to the P loop of the voltage-activated Ca^2+^, NA^2+^, and K^2+^ channels, was located in the loop region between the putative fifth and sixth transmembrane helices in TciRyR. Two Ca^2+^-binding EF-hands (calcium-binding motif) motifs originally reported in the lobster RyR were also present in tandem at positions 4186–4214 and 4223–4249 in TciRyR (Figure 3). A glutamate residue likely involved in the Ca^2+^ sensitivity in rabbit RyR3 (E^3885^) and RyR1 (E^4032^) was also detected in TciRyR (E^4145^). Additionally, residues corresponding to I^4897^, R^4913^, and D^4917^ in rabbit RyR1, which were shown to play an important role in the activity and conductance of the Ca^2+^ release channel, were also well conserved in TciRyR (I^4958^, R^4974^, D^4978^) (Figure 3).

### 2.4. mRNA Expression Profiles of TciRyR

**Figure 4:**
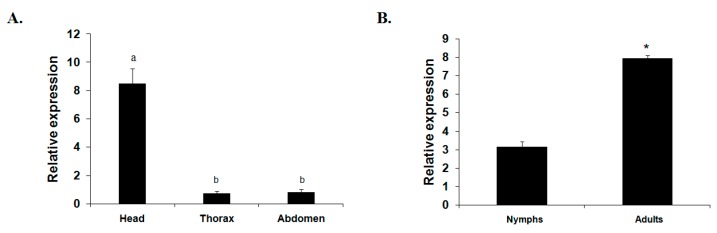
The relative mRNA expression profiles of *TciRyR*. (**A**) Expression profiles in different adult body parts including the head, thorax, and abdomen; (**B**) Expression profiles during different developmental stage including nymph and adult. Different letters above each bar indicate statistical difference by ANOVA (* *p* <0.05).

To understand the developmental and body parts’ expression profiles of *TciRyR*, the mRNA levels of this gene were analyzed using RT-qPCR in nymph, adult, adult head, adult thorax, and adult abdomen of *T. citricida* (Figure 4). The expression pattern revealed that *TciRyR* was expressed in both of the tested developmental stages and body parts. The expression levels of *TciRyR* in adults were significant higher than those in nymphs. Significantly higher expression was observed in the adult heads than in the thoraxes and abdomens.

### 2.5. Alternative Splicing of TciRyR

**Figure 5:**
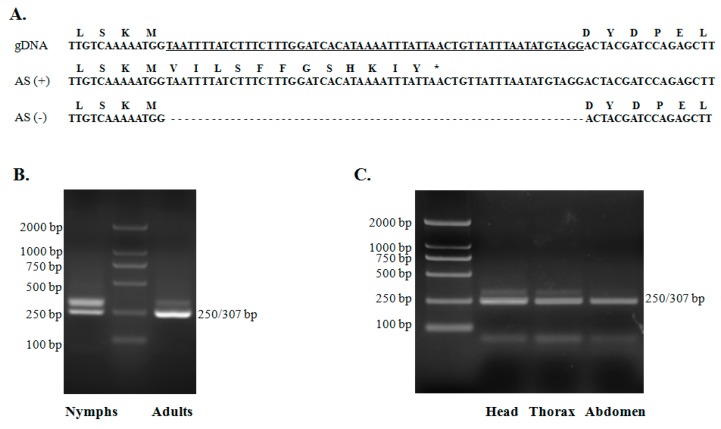
Alternative exon in *TciRyR*. (**A**) Nucleotide and inferred amino acid sequences of alternative exons in the *TciRyR* gene. Underlined sequence indicates the intron; (**B**) Semi-qRT-PCR detection of AS in nymphs and adults stages; (**C**) Semi-qRT-PCR detection of alternatively spliced (AS) in adult head, adult thorax, and adult abdomen. Different bands indicate the presence or absence of specific exons in the various PCR products. Semi-qRT-PCR products were separated on a 2% agarose gel.

Aligning multiple cDNA clones revealed the presence of alternative splicing variants in *TciRyR*, leading to the discovery of one alternatively spliced (AS) exon in *TciRyR* (Figure 1 and Figure 5). The splicing site was located at position 2780–2789, close to the third RyR domain. Further RT-PCR and sequencing analysis confirmed that this 57 bp exon was optional (Figure 5A). The genome organization of this region indicated that this alternative exon was derived from intron retention, inclusion of which could lead to the production of truncated proteins (Figure 5A).

**Figure 6:**
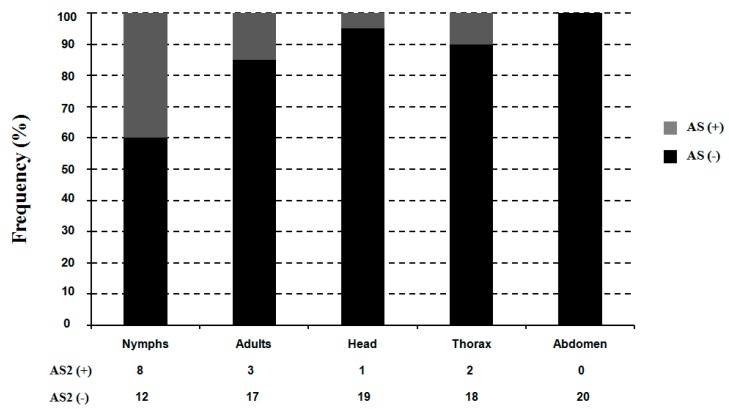
The relative frequencies of the *TciRyR* optional exon (AS) in nymph, adult, adult head, adult thorax, and adult abdomen. The number of clones containing each alternative splice variant amplified by semi-qRT PCR is listed below.

The splicing frequency at this site was determined by diagnostic PCR for the five mRNA samples (nymph, adult, adult head, adult thorax, and adult abdomen) (Figure 6). Data were collected from 20 clones from each sample. Variants retaining the alternative exon were detected at lower frequencies compared with those with the alternative exon spliced out. The highest frequency (40%) of the intron retention was observed in the nymph, followed by adults (15%), head (5%), thorax (10%) and abdomen (0%) (Figure 6).

## 3. Discussion

RyRs have been investigated extensively in mammals and the current knowledge on insect RyRs are mostly derived from studies on mammalian RyRs. Since the discovery of the first insect RyR from *D. melanogaster* in 1994 [10], only 15 insect RyR orthologs have been reported thus far [17,18,19,20,21,22,23,24,25,26,27,28], despite the increasing recognition of great potential of diamide insecticides in insect control and the importance of the molecular information of RyRs to new insecticides design. Now that the molecular structure of RyRs accounts for their affinity with the drugs and functional mechanisms [33], isolating the full-length RyR cDNA is a critical step towards a comprehensive understanding of the function of RyRs. In the present study, a new RyR ortholog was identified from an economically important citrus pest, *T. citricida*, by providing complete RyR coding information. This information would be crucial for us to understand the molecular basis responsible for the affinity difference of various RyRs with the diamide insecticides.

Like other insect RyRs, TciRyR was also a very large molecule, with a theoretical molecular weight of 580.08 kDa, putatively encoded by 15,306 nucleotides. Such high molecular weight may be one of the reasons to hamper the increasing records of RyR members with full-length cDNAs. TciRyR shared over 70% identity with the RyRs of other insect species, suggesting that insect RyRs are evolutionarily conserved, which is in accordance with the published results about RyRs [17,18,19,20,21]. Insect RyRs shared relatively low similarity with the mammalian RyR isoforms [10], which were also confirmed by our results, that 43% to 46% identity between TciRyR with the mammalian homologs was observed. The result was also supported by phylogenetic analysis, which indicated that all insect RyRs (*n* = 36) clustered into a large branch, which was well segregated from the RyRs from Crustacea, Arachnida, Nematode, and Mammal. These results also corroborate the great potential of insect RyRs as insecticide targets taking into account the low similarity with their mammalian homologs.

The typical features crucial for RyRs were conserved in TciRyR, including one MIR domain, which has a ligand transferase function [34], two RIH domains, which form the IP_3_ binding site together with the MIR domain in IP_3_Rs [35], three SPRY domains, which are known to mediate protein-protein interactions [36], and four RyR repeat domains, which are unique to RyR channels and conserved in all the members of the intracellular Ca^2+^ release channel superfamily [37]. In addition, TciRyR also had the well conserved sequence motif, GXRXGGGXGD (GVRAGGGIGD), which constitutes part of the pore-forming segments of the Ca^2+^ release channels [38], implying that TciRyR was likely to form a functional Ca^2+^-selective channel. Phosphorylation has been known to play important roles in modulating the functional properties of proteins [34]. Hyper-phosphorylation of the cardiac Ca^2+^ release channel (RyR2) by PKA at serine-2808 has been proposed to be a key mechanism for cardiac dysfunction in heart failure [39]. Three potential PKA and ten potential PKC sites were present in TciRyR, which implicated that TciRyR may also undergo abundant post-translation regulation.

The differential expression levels of genes in different developmental stages or body parts suggested that different functions might be involved. In this study, expression analysis of *TciRyR* revealed the highest mRNA expression levels were observed in the adult and especially in head. Similar results were also reported in *H. armigera*, *P. xylostella* and *L. decemlineata* [20,22,25]. Because the head is critical for hormone secretion and nerve conduction, these results may be associated with the neuronal calcium signal in the neuronal system.

Alternative splicing of pre-mRNA transcripts is a prevalent feature of gene processing and is critical for protein diversity [40]. More than 10 distinct splicing variants of RyRs have been identified in human, rabbit, mouse, mink, and dog [40]. Some splice variants of RyR isoforms were found to predominantly suppress Ca^2+^ release or contribute to distinct Ca^2+^ releasing patterns [40,41,42]. Similarly, alternative splicing also exists in insect RyRs. Previously, it has been shown that *MpRyR* did not have alternative splicing at any developmental stages likely due to the asexual mode of *M. persicae* reproduction [24]. Interestingly, *TciRyR* underwent splicing by intron retention, a relative rare splicing event in animals, which lead to the generation of an optional exon (Figure 5). However, the inclusion of this exon induced a premature stop codon intro TciRyR, which only encoded truncated proteins. To the best of our knowledge, no reports have ever confirmed that other insect RyRs underwent such a splicing event, which could lead to the production of truncated proteins. This has led us to doubt whether such splicing events results from biological errors, and after all the truncated proteins have lost some of the key regions required for functional RyRs, such as the pore-helix and the transmembrane segments. Thus, we further examine the splicing patterns in different stages and body parts. The results indicated that such a splicing event was abundant in most of the samples, except in the abdomen (Figure 5 and Figure 6), and 40% of the splicing variants were determined to contain the splicing exons. These results suggested that this splicing event was developmentally regulated, supporting that such truncated proteins did exist and might perform certain biological functions. More information is required to determine whether this splicing event was *T. citricida-*specific or widely exited in other species.

## 4. Experimental Section

### 4.1. Insects

The brown citrus aphids were collected from the citrus orchard in the experimental farm at Southwest University, Chongqing, China, in 2014, and were maintained in the laboratory on the fresh citrus seedlings without exposure to pesticides at 25 ± 1°C, 75%–80% relative humidity and 14:10 h light:dark photoperiod.

### 4.2. RNA Extraction and cDNA Synthesis

Total RNA were isolated from the whole body of *T. citricida* using the RNeasy plus Micro kit (Qiagen GmbH, Hilden, Germany) following the manufacturer’s instructions. RNA were quantified by measuring the absorbance at 260 nm using a NanoVue UV–Vis spectrophotometer (GE Healthcare Bio-Science, Uppsala, Sweden). The purity of all RNA samples were assessed from the absorbance ratio at OD260/280 and OD260/230, and RNA integrity was checked on a 1.0% agarose gel by electrophoresis. The first strand cDNA was synthesized from 2 μg of each total RNA preparation using PrimeScript™ II 1st Strand cDNA Synthesis Kit (Takara, Dalian, China) according to the manufacturer’s instructions.

### 4.3. Cloning of RyR Genes from T. citricida

Nine putative short cDNA fragments encoding *TciRyR* were retrieved from the *T. citricida* transcriptome [43]. Based on these nine fragment and the highly conserved domains of the RyR gene from *Acyrthosiphon pisum* (*ApRyR*) (GenBank accession number: XP_003246190), gene-specific primers (Table S1) were designed using Primer Premier 5.0 (Premier Biosoft International, Palo Alto, CA, USA) and DNAMAN v.6.03 (Lynnon Biosoft, San Ramon, CA, USA) to amplify overlapping cDNA fragments. PCR was carried out with rTaq™ polymerase (TaKaRa, Dalian, China) in a 25 μL reaction volume containing 2.5 μL Buffer (Mg^2+^ free), 2.5 μL Mg^2+^ (25 mM), 2 μL dNTPs (2.5 mM), 1 μL cDNA template and 1 μL of each gene-specific primer (10 μM). Thermal cycling conditions were as follows: initial denaturation at 95 °C for 3 min; followed by 35 cycles of denaturation at 95 °C for 30 s, annealing at 55–65 °C (based on the primer annealing temperatures) for 30 s, and extension at 72 °C for 2–3 min (based on the size of expected fragment); and a final extension at 72 °C for 10 min. The amplified cDNA fragments were separated by 1.0% agarose gel electrophoresis and purified with a Gel Extraction Mini Kit (TakaRa, Dalian, China). The purified products were cloned into the pGEM-T Easy vector (Promega, Madison, WI, USA) and transformed into chemically competent *Trans5α* cells (TransGen, Beijing, China). Finally, recombinant plasmids were sequenced from both directions using an automated ABI Model 3100 sequencer (Life Technologies, Shanghai, China).

### 4.4. Sequence Analysis and Phylogenetic Tree Construction

The complete coding regions of *TciRyR* were obtained by assembling ten overlapping PCR products using DNAMAN v.6.03 (Lynnon Biosoft). Multiple sequence alignments of the deduced amino acid sequences were performed by the online server Clustal W2 [44]. The theoretical isoelectric point and molecular weights were predicted using ExPASy Proteomics Server [45]. Transmembrane segments were predicted using the TMHMM server 2.0 [46]. Conserved domains were predicted using the Conserved Domains Database [47]. Protein kinase A (PKA) and protein kinase C (PKC) sites were predicted by KinasePhos [48], and *N*-Glycosylation sites were predicted by the NetNGlyc 1.0 server [49]. The RyR homologs used in the phylogenetic analysis were retrieved from GenBank [50], by using “ryanodine receptor” as the key words. The phylogenetic tree was constructed by maximum likelihood using MEGA6.0 [51], and the Jones-Taylor-Thornton (JTT) for amino acid substitution model was used. Test of phylogeny was performed by bootstrap method with 1000 replications.

### 4.5. Quantitative Real-Time PCR

Quantitative RT-PCR (RT-qPCR) was used to examine the relative mRNA levels of *TciRyR* in five different samples: nymph, adult, adult head, adult thorax, and adult abdomen. The RT-qPCR was performed in 10 μL reaction mixtures containing 5 μL GoTaq^®^qRCR Mix (Promega), 3 μL ddH_2_O, 0.5 μL of each primer (10 mM), and 1 μL of template cDNA, following the common cycle: pre-incubation at 95 °C for 2 min, 40 cycles of denaturation at 95 °C for 15 s, annealing at 60 °C for 30 s, extension at 72 °C for 30 s. After each reaction, a melting curve analysis from 60 to 95 °C was performed to ensure consistency and the specificity of the amplified product. The primers used in RT-qPCR (Table S1) were designed using Primer 3 (version 0.4.0) software [52]. The normalization of the expression levels was performed using the reference gene *Elongation factor-1alpha* (EF-1α) (Table S1). Relative expression levels were analyzed using the 2^−ΔΔ*C*t^ method [53]. Significant difference was tested by One-way analysis of variance (ANOVA) for multiple sample comparisons with SPSS17.0 software (SPSS Inc., Chicago, IL, USA), and a value of *p* < 0.05 was considered to be statistically significant.

### 4.6. Diagnostic PCR Analysis

Diagnostic PCR was performed to detect the presence of each putative alternative exon in the individual cDNA clone. Briefly, segments containing the alternative exons were amplified by primers flanking the splicing region (Table S1). The PCR reaction system and conditions were the same as mentioned above. To detect the presence of alternative exons, PCR products were separated on a 2.0% agarose gel and visualized using a Gel Doc XR+System (BIO-RAD, Hercules, CA, USA). The presence or absence of an alternative exon was indicated by the band patterns, and these amplified PCR products were cloned and sequenced essentially as described above.

Genomic DNA of *T. citricida* was extracted from 30 adult heads using a Tissue/cell gDNA Mini Kit (Promega, Madison, WI, USA) following the manufacturer’s protocols. Primers flanking the alternative exons were same as those used in the diagnostic PCR to determine the genomic organization of splicing site. PCR was performed the same as mentioned above.

## 5. Conclusions

In summary, this study provided the complete coding sequences and determined the expression patterns of RyR from the damaging citrus pests and disease vectors, *T. citricida*. We also confirmed that TciRyR underwent alternative splicing, which implicates that more research is needed to reveal the mRNA diversity, putatively responsible for the functional diversity. On the one hand, our results about TciRyR could not only enrich our current knowledge about insect RyRs, but also contribute to estimating the potential of the newly developed diamide insecticides in controlling aphids. On the other hand, now that the RyRs were widely distributed in the animal kingdom as shown in our investigation, our information about TciRyR is of great importance for development of new insecticides, selectively acting on the target pests.

## Supplementary Materials

Supplementary materials can be found at http://www.mdpi.com/1422-0067/16/07/15220/s1.

## References

[1] Fill M., Copello J.A. (2002). Ryanodine receptor calcium release channels. Physiol. Rev..

[2] Berridge M.J. (1993). Inositol trisphosphate and calcium signaling. Nature.

[3] Clapham D.E. (1995). Calcium signaling. Cell.

[4] Takeshima H. (1993). Primary structure and expression from cDNAs of the ryanodine receptor. Ann. N. Y. Acad. Sci..

[5] Wagenknecht T., Grassucci R., Frank J., Saito A., Inui M., Fleischer S. (1989). Three dimensional architecture of the calcium channel/foot structure of sarcoplasmic reticulum. Nature.

[6] Bhat M.B., Zhao J., Takeshima H., Ma J. (1997). Functional calcium release channel formed by the carboxyl-terminal portion of ryanodine receptor. Biophys. J..

[7] Otsu K., Willard H.F., Khanna V.K., Zorzato F., Green N.M., MacLennan D.H. (1990). Molecular cloning of cDNA encoding the Ca^2+^ release channel (ryanodine receptor) of rabbit cardiac muscle sarcoplasmic reticulum. J. Biol. Chem..

[8] Ogawa Y., Murayama T., Kurebayashi N. (1999). Comparison of properties of Ca^2+^ release channels between rabbit and frog skeletal muscles. Mol. Cell. Biochem..

[9] Ottini L., Marziali G., Conti A., Charlesworth A., Sorrentino V. (1996). Alpha and beta isoforms of ryanodine receptor from chicken skeletal muscle are the homologues of mammalian RyR1 and RyR3. Biochem. J..

[10] Takeshima H., Nishi M., Iwabe N., Miyata T., Hosoya T., Masai I., Hotta Y. (1994). Isolation and characterization of a gene for a ryanodine receptor/calcium release channel in *Drosophila melanogaster*. FEBS Lett..

[11] Xu X., Bhat M.B., Nishi M., Takeshima H., Ma J. (2000). Molecular cloning of cDNA encoding a *Drosophila* ryanodine receptor and functional studies of the carboxyl-terminal calcium release channel. Biophys. J..

[12] Tohnishi M., Nakao H., Furuya T., Seo A., Kodama H. (2005). Flubendiamide, a novel insecticide highly active against Lepidopterous insect pests. J. Pestic. Sci..

[13] Lahm G.P., Selby T.P., Freudenberger J.H., Stevenson T.M., Myers B.J. (2005). Insecticidal anthranilic diamides: A new class of potent ryanodine receptor activators. Bioorg. Med. Chem. Lett..

[14] Pepper B.P., Carruth L.A. (1945). A new plant insecticide for control of the European corn borer. J. Econ. Entomol..

[15] Li X.C., Degain B.A., Harpold V.S., Marcon P.G., Nichols R., Fournier A.J., Naranjo S.E., Palumbo J.C., Ellsworth P.C. (2012). Baseline susceptibilities of B- and Q-biotype *Bemisia tabaci* to anthranilic diamides in Arizona. Pest Manag. Sci..

[16] Zhang R.M., Jiang E.B., He S.Y., Chen J.H. (2015). Lethal and sublethal effects of cyantraniliproleon *Bactrocera dorsalis* (Hendel) (Diptera: Tephritidae). Pest Manag. Sci..

[17] Wang J., Li Y., Han Z., Zhu Y., Xie Z. (2012). Molecular characterization of a ryanodine receptor gene in the rice leaffolder, *Cnaphalocrocis medinalis* (Guenee). PLoS ONE.

[18] Wang J., Xie Z., Gao J., Liu Y., Wang W. (2014). Molecular cloning and characterization of a ryanodine receptor gene in brown plant hopper (BPH), *Nilaparvata lugens* (Stal). Pest Manag. Sci..

[19] Yuan G.R., Shi W.Z., Yang W.J. (2014). Molecular characteristics, mRNA expression, and alternative splicing of a ryanodine receptor gene in the Oriental Fruit Fly, *Bactrocera dorsalis* (Hendel). PLoS ONE.

[20] Wang X., Wu S., Yang Y., Wu Y. (2012). Molecular cloning, characterization and mRNA expression of a ryanodine receptor gene from diamondback moth, *Plutella xylostella*. Pestic. Biochem. Physiol..

[21] Cui L., Yang D., Yan X., Rui C., Wang Z. (2013). Molecular cloning, characterization and expression profiling of a ryanodine receptor gene in Asian corn borer, *Ostrinia furnacalis* (Guenee). PLoS ONE.

[22] Wang J., Liu Y., Gao J., Xie Z., Huang L. (2013). Molecular cloning and mRNA expression of a ryanodine receptor gene in the cotton bollworm, *Helicoverpa armigera*. Pestic. Biochem. Physiol..

[23] Wu S., Wang F., Huang J., Fang Q., Shen Z. (2013). Molecular and cellular analyses of a ryanodine receptor from hemocytes of *Pieris rapae*. Dev. Comp. Immunol..

[24] Troczka B.J., Williams A.J., Bass C. (2015). Molecular cloning, characterisation and mRNA expression of the ryanodine receptor from the peach-potato aphid, *Myzus persicae*. Gene.

[25] Wan P.J., Guo W.Y., Yang Y. (2014). RNAi suppression of the ryanodine receptor gene results in decreased susceptibility to chlorantraniliprole in Colorado potato beetle *Leptinotarsa decemlineata*. J. Insect Physiol..

[26] Yang Y., Wan P.J., Hu X.X. (2014). RNAi mediated knockdown of the ryanodine receptor gene decreases chlorantraniliprole susceptibility in *Sogatella furcifera*. Pestic. Biochem. Physiol..

[27] Liu Y.P., Li C.J., Gao J.K., Wang W.L., Li H., Guo X.Z., Li B., Wang J.J. (2014). Comparative characterization of two intracellular Ca^2+^-release channels from the red flour beetle, *Tribolium castaneum*. Sci. Rep..

[28] Liu Y.L., Shahzad M.F., Zhang L., Li F., Lin K.J. (2013). Amplifying long transcripts of ryanodine receptors of five agricultural pests by transcriptome analysis and gap filling. Genome.

[29] Sattelle D.B., Cordova D., Cheek T.R. (2008). Insect ryanodine receptors: molecular targets for novel pest control chemicals. Invertebr. Neurosci..

[30] Troczka B., Zimmer C.T., Elias J., Schorn C., Bass C., Davies T.G. (2012). Resistance to diamide insecticides in diamondback moth, *Plutella xylostella* (Lepidoptera: Plutellidae) is associated with a mutation in the membrane-spanning domain of the ryanodine receptor. Insect Biochem. Mol. Biol..

[31] Guo L., Wang Y., Zhou X. (2014). Functional analysis of a point mutation in the ryanodine receptor of *Plutella xylostella* (L.) associated with resistance to chlorantraniliprole. Pest Manag. Sci..

[32] Guo L., Pei L., Zhou X.G., Gao X.W. (2014). Novel mutations and mutation combinations of ryanodine receptor in a chlorantraniliprole resistant population of *Plutella xylostella* (L.). Sci. Rep..

[33] Yan Z., Bai X.C., Yan C.Y., Wu J.P., Li Z.Q., Xie T., Peng W., Yin C.C., Li X.M., Scheres H.W. (2015). Structure of the rabbit ryanodine receptor RyR1 at near-atomic resolution. Nature.

[34] Ikenoue T., Inoki K., Yang Q. (2008). Essential function of TORC2 in PKC and Akt turn motif phosphorylation, maturation and signaling. EMBO J..

[35] Ponting C.P. (2000). Novel repeats in ryanodine and IP3 receptors and protein *O*-mannosyltransferases. Trends Biochem. Sci..

[36] Cui Y., Tae H.S., Norris N.C., Karunasekara Y., Pouliquin P., Board P.G., Dulhunty A.F., Casarotto M.G. (2009). A dihydropyridine receptor α_1s_ loop region critical for skeletal muscle contraction is intrinsically unstructured and binds to a SPRY domain of the type 1 ryanodine receptor. Int. J. Biochem. Cell Biol..

[37] Sorrentino V., Barone V., Rossi D. (2000). Intracellular Ca^2+^ release channels in evolution. Curr. Opin. Genet. Dev..

[38] Zhao M., Li P., Li X. (1999). Molecular identification of the ryanodine receptor pore-forming segment. J. Biol. Chem..

[39] Xiao B., Jiang M.T., Zhao M. (2005). Characterization of a novel PKA phosphorylation site, serine-2030, reveals no PKA hyperphosphorylation of the cardiac ryanodine receptor in canine heart failure. Circ. Res..

[40] George C.H., Rogers S.A., Bertrand B.M.A., Tunwell R.E.A., Thomas N.L. (2007). Alternative splicing of ryanodine receptors modulates cardiomyocyte Ca^2+^ signaling and susceptibility to apoptosis. Circ. Res..

[41] Kimura T., Lueck J.D., Harvey P.J., Pace S.M., Ikemoto N. (2009). Alternative splicing of RyR1 alters the efficacy of skeletal EC coupling. Cell Calcium.

[42] Takasawa S., Kuroki M., Nata K., Noguchi N., Ikeda T. (2010). A Novel ryanodine receptor expressed in pancreatic islets by alternative splicing from type 2 ryanodine receptor gene. Biochem. Biophys. Res. Commun..

[43] Wang J.J. (2015).

[44] Larkin M., Blackshields G., Brown N., Chenna R., McGettigan P.A., McWilliam H. (2007). Clustal W and Clustal X version 2.0. Bioinformatics.

[45] Gasteiger E., Hoogland C., Gattiker A., Duvaud S., Wilkins M.R., Appel R.D., Bairoch A. (2005). Protein identification and analysis tools on the ExPASy server. Proteomics Protoc. Handb..

[46] Krogh A., Larsson B., von Heijne G., Sonnhammer E.L. (2001). Predicting transmembrane protein topology with a hidden Markov model: Application to complete genomes. J. Mol. Biol..

[47] Marchler-Bauer A., Derbyshire M.K., Gonzales N.R., Lu S., Chitsaz F., Geer L.Y., Geer R.C., He J., Gwadz M., Hurwitz D.I. (2015). CDD: NCBI’s conserved domain database. Nucleic Acids Res..

[48] Huang H.D., Lee T.Y., Tseng S.W. (2005). KinasePhos: A web tool for identifying protein kinase-specific phosphorylation sites. Nucleic Acids Res..

[49] Blom N., Sicheritz-Pontén T., Gupta R. (2004). Prediction of post-translational glycosylation and phosphorylation of proteins from the amino acid sequence. Proteomics.

[50] Benson D.A., Clark K., Karsch-Mizrachi I., Lipman D.J., Ostell J., Sayers E.W. (2015). GenBank. Nucleic Acids Res..

[51] Tamura K., Stecher G., Peterson D., Filipski A., Kumar S. (2013). MEGA6: Molecular evolutionary genetics analysis version 6.0. Mol. Biol. Evol..

[52] Untergrasser A., Cutcutache I., Koressaar T., Ye J., Faircloth B.C., Remm M., Rozen S.G. (2012). Primer 3—New capabilities and interfaces. Nucleic Acids Res..

[53] Kenneth L.J., Thomas S.D. (2001). Analysis of relative gene expression data using real-time quantitative PCR and the 2^−ΔΔ*C*t^ method. Methods.

