# Supplementary material for: Molecular Characterization, mRNA Expression and Alternative Splicing of Ryanodine Receptor Gene in the Brown Citrus Aphid, Toxoptera citricida (Kirkaldy)
Source: Int J Mol Sci. 2015 Jul 6;16(7):15220–34. doi: 10.3390/ijms160715220 (PMC4519896; doi:10.3390/ijms160715220)
Supplement: Supplementary file 1 [file ijms-16-15220-s001.pdf]

Supplementary Information

Table S1. Primers used for RT-PCR, qRT-PCR and diagnostic PCR in *TciRyR*.

Description (cDNA Position)	Primer Name	Sequence (5′ to 3′)
RT-PCR product Tci1 (1–237)	TciRyR1.F	TATTCGCAAACGGACAGAGAC
	TciRyR1.R	TGCTCCACGTCGACGGTAT
RT-PCR product Tci2 (206–659)	TciRyR2.F	CAGTAGAATGAAATACGTTGGTT
	TciRyR2.R	TTCGATAAACGGCAGATCCAC
RT-PCR product Tci3 (616–1240)	TciRyR3.F	TGTAGCCGTAAGAAGTTCACA
	TciRyR3.R	CGTAAACATGAAGAGACCGGTAA
RT-PCR product Tci4 (1190–1641)	TciRyR4.F	TCAAGGAGAAGGATTTGTACC
	TciRyR4.R	GTAAATGACATCACTCCAGTAGC
RT-PCR product Tci5 (1600–2059)	TciRyR5.F	AAAGCTGTTACATGGTTCGATC
	TciRyR5.R	AGGTGGACATCGGAACTCTCTC
RT-PCR product Tci6 (1966–2581)	TciRyR6.F	GAAACTTGGGATCCTGAATTTCA
	TciRyR6.R	AGGGCATATCACTCTTTGGTCG
RT-PCR product Tci7 (2553–3105)	TciRyR7.F	ACTTGAAGATCTACACGGTGTT
	TciRyR7.R	CTAACGGTAGTACAACTTTGGAG
RT-PCR product Tci8 (3063–3731)	TciRyR8.F	TGATTCATTATATTGACAGAGCG
	TciRyR8.R	ATAAGTCGCCTTGCTTTGAAG
RT-PCR product Tci9 (3700–4406)	TciRyR9.F	AGTTGATGAGGTAGCGGATAGAA
	TciRyR9.R	AGCCTGTATGCCTCTTCGAAT
RT-PCR product Tci10 (4386–5101)	TciRyR10.F	CTTATCTTGCAGACGACGAA
	TciRyR10.R	ATATGATTCGCTTCTACTGAG
Diagnostic PCR for exon AS	AS.F	ATTACGATCGATGTGCAAAGTAC
	AS.R	TTCTGTTTCGAACCATACCACTC
qRT-PCR	qRyR.F	GATCTCCGCCAAGTCGTTTC
	qRyR.R	TCGTGACTCCAGCTTTCCAT
Elongation factor-1alpha	EF-1α.F	GATGCACCTGGTCACAGAGA
	EF-1α.R	CCATCTTGTTCACACCAACG

Table S2. RyRs used in phylogenetic analysis.

RyR	Species	GenBank Accession Number	Length (bp)	PI	Molecular Weight (kDa)
DmRyR	*Drosophila melanogaste*	BAA41470	5126	5.14	580.46
DgRyR	*Drosophila grimshaw*	XP_001995333	5174	5.38	585.68
CcyRyR	*Ceratitis capitata*	XP_004527515.1	5117	5.38	579.74
BdRyR	*Bactrocera dorsalis*	AHY02115.1	5140	5.38	582.29
MdRyR	*Musca domestica*	XP_005177534.1	5111	5.42	579.39
DmoRyR	*Drosophila mojavensis*	XP_002005714	5175	5 38	586.16
AgRyR	*Anopheles gambiae*	XP_318561	5109	5.33	577.54
AdRyR	*Anopheles darling*	ETN61080.1	5004	5.33	565.87
AaRyR	*Aedes aegypti*	XP_001657320	5118	5.33	578.55
sRyR	*Bombyx mori*	DJ085056	5105	5.44	577.20
PxRyR	*Plutella xylostella*	AET09964.1	5164	5.48	583.73
HaRyR	*Helicoverpa armigera*	AHB33498.1	5142	5.47	581.09
PrRyR	*Pieris rapae*	AGI62938.1	5106	5.46	578.12

Table S2.*Cont.*

RyR	Species	GenBank Accession Number	Length (bp)	PI	Molecular Weight (kDa)
OfRyR	*Ostrinia furnacalis*	AGH48757.1	5108	5.42	577.49
CsRyR	*Chilo suppressalis*	AFN70719.1	5128	5.43	580.32
BtRyR	*Bemisia tabaci*	AFK84957	5142	5.47	580.82
NlRyR	*Nilaparvata lugens*	KF306296	5135	5.36	579.78
LsRyR	*Laodelphax striatellus*	AFK84959	5115	5.36	578.65
SfRyR	*Sogatella furcifera*	AHW99829.1	5128	5.36	579.14
MpRyR	*Myzus persicae*	AJA41114.1	5101	5.48	579.98
ApRyR	*Acyrthosiphon pisum*	XP_003246190	5101	5.46	579.96
TiRyR	*Triatoma infestans*	JAC16718.1	5065	5.38	572.40
AmRyR	*Apis mellifera*	XP_392217	5102	5.38	578.67
AdRyR	*Apis dorsata*	XP_006622367.1	5106	5.38	579.17
AfRyR	*Apis florae*	XP_003696991.1	5023	5.44	570.27
NvRyR	*Nasonia vitripennis*	XP_008202582.1	5130	5.36	582.32
BiRyR	*Bombus impatiens*	XP_003393894.1	5108	5.37	579.49
MrRyR	*Megachile rotundata*	XP_003701507	5084	5.42	576.86
HsaRyR	*Harpegnathos saltator*	EFN78897.1	5080	5.42	575.94
CbRyR	*Cerapachys biroi*	EZA52107.1	5061	5.47	573.89
AeRyR	*Acromyrmex echinatior*	XP_011054963.1	5096	5.43	577.97
LdRyR	*Leptinotarsa decemlineata*	AHW99830.1	5128	5.53	581.61
TcRyR	*Tribolium castaneum*	AIU40166.1	5094	5.39	577.09
DpRyR	*Dendroctonus ponderosae*	ENN70900.1	4928	5.39	559.55
TuRyR	*Tetranychus urticae*	BAK26392.1	5180	5.54	583.55
MoRyR	*Metaseiulus occidentalis*	XP_003748443.1	5162	5.33	581.54
CsaRyR	*Cupiennius salei*	JAA92932.1	5056	5.27	570.77
DpuRyR	*Daphnia pulex*	EFX89429.1	5119	5.45	577.95
CbriRyR	*Caenorhabditis briggsae*	XP_002637345	4183	5.47	476.29
CeRyR	*Caenorhabditis elega*	BAA08309	5071	5.48	576.36
CrRyR	*Caenorhabditis remanei*	EFP05547	5220	5.59	593.20
CbreRyR	*Caenorhabditis brenneri*	EGT47004	5370	5.61	610.85
AmRyR1	*Ailuropoda melanoleuca RyR1*	XP_002925900	4841	5.20	545.98
AmRyR2	*Ailuropoda melanoleuca RyR2*	XP_002923703	4914	5.73	558.09
AmRyR3	*Ailuropoda melanoleuca RyR3*	XP_002925256	4855	5.67	550.31
HsRyR1	*Homo sapiens RyR1*	NP_000531	5038	5.18	565.18
HsRyR2	*Homo sapiens RyR2*	NP_001026	4967	5.73	564.57
HsRyR3	*Homo sapiens RyR3*	NP_001027	4870	5.47	552.04
OcRyR1	*Oryctolagus cuniculus RyR1*	NP_001095188	5037	5.16	565.25
OcRyR2	*Oryctolagus cuniculus RyR2*	NP_001076226	4968	5.70	565.09
OcRyR3	*Oryctolagus cuniculus RyR3*	NP_001076231	4872	5.58	551.93
